# An Agent-Based Model to Reproduce the Boolean Logic Behaviour of Neuronal Self-Organised Communities through Pulse Delay Modulation and Generation of Logic Gates

**DOI:** 10.3390/biomimetics9020101

**Published:** 2024-02-09

**Authors:** Luis Irastorza-Valera, José María Benítez, Francisco J. Montáns, Luis Saucedo-Mora

**Affiliations:** 1 E.T.S. de Ingeniería Aeronáutica y del Espacio, Universidad Politécnica de Madrid, Pza. Cardenal Cisneros 3, 28040 Madrid, Spain; luis.irastorza_valera@ensam.eu (L.I.-V.); josemaria.benitez@upm.es (J.M.B.); fco.montans@upm.es (F.J.M.); 2 PIMM Laboratory, Arts et Métiers Institute of Technology, 151 Bd de l’Hôpital, 75013 Paris, France; 3 Department of Mechanical and Aerospace Engineering, Herbert Wertheim College of Engineering, University of Florida, Gainesville, FL 32611, USA; 4 Department of Materials, University of Oxford, Parks Road, Oxford OX1 3PJ, UK; 5 Department of Nuclear Science and Engineering, Massachusetts Institute of Technology, Cambridge, MA 02139, USA

**Keywords:** connectome, graph theory, mathematical modelling, agent-based modelling, metastability, backpropagation, neuroplasticity, neuronal migration, computational neuroscience

## Abstract

The human brain is arguably the most complex “machine” to ever exist. Its detailed functioning is yet to be fully understood, let alone modelled. Neurological processes have logical signal-processing and biophysical aspects, and both affect the brain’s structure, functioning and adaptation. Mathematical approaches based on both information and graph theory have been extensively used in an attempt to approximate its biological functioning, along with Artificial Intelligence frameworks inspired by its logical functioning. In this article, an approach to model some aspects of the brain learning and signal processing is presented, mimicking the metastability and backpropagation found in the real brain while also accounting for neuroplasticity. Several simulations are carried out with this model to demonstrate how dynamic neuroplasticity, neural inhibition and neuron migration can reshape the brain’s logical connectivity to synchronise signal processing and obtain certain target latencies. This work showcases the importance of dynamic logical and biophysical remodelling in brain plasticity. Combining mathematical (agents, graph theory, topology and backpropagation) and biomedical ingredients (metastability, neuroplasticity and migration), these preliminary results prove complex brain phenomena can be reproduced—under pertinent simplifications—via affordable computations, which can be construed as a starting point for more ambitiously accurate simulations.

## 1. Introduction

Studying the brain’s structure is a difficult task for multiple reasons, tackled from very different perspectives [1]. There is no univocal model or chart of the brain because of the individual variability, which is not necessarily caused by pathologies. This variability makes the overall description of the brain and its standardization on the micro- and nano-scales challenging. Furthermore, obtaining measurements through microscopy presents its own difficulties: tissue handling, contrast, stain density, dissection, lighting, etc. [2].

MRI (Magnetic Resonance Imaging) has made the task of studying the brain structure easier, with a greater resolution [3] and less risk than radiation-based methodologies such as X-rays, Computerized Tomography (CT-scans) or Positron Emission Tomography (PET). However, MRI is, in general, contraindicated for patients with implants or pacemakers, limiting its applications. The more recent MRI functional variant (fMRI) leverages changes in blood flow associated with brain activity, obtaining some promising global brain mappings [4,5,6,7,8].

It is used prior to surgery and is key in bringing together different time and spatial scales in the brain [9] and to exploring feedback and feed-forward behaviour within cortical layer hierarchy [10]. Alas, the technique is also conditioned by task execution, signal-to-noise ratio and patient comorbidity [11]. There are other techniques used to understand the structure and functioning of the brain like Diffusion Tensor Imaging (DTI) and Transcranial Magnetic Stimulation (TMS).

Zooming into the nano-scale, the brain’s most basic individual parts are neurons: electrically excitable cells allowing for the transmission of information through the nervous system. Neurons have various shapes and specific functions, but they do usually share a basic structure composed of dendrites conveying information from preceding neurons into a nucleus (soma), which sends signals along an axon—enveloped to varying degrees by a myelin sheath—onto the next one. Measuring a neuron’s activity or inactivity is a complex task which is subject to morphological and method-driven variations.

Neuron counting has evolved from microscopical measures [12]—with the help of MRI [13], stereology [14,15] or cytometry [16]—to state-of-the-art solutions involving Deep Learning [17]; whereas tracking individual neuronal activity necessarily implies local measurement of biological and/or electrical indicators. Examples of targeted biological indicators are proteins such as PSD-95, whose decay is linked to Alzheimer’s disease [18,19,20,21]. Electrical indicators are obtained through electrophysiological studies [22,23].

This article presents a different approach to study brain functioning and, in particular, damage-related changes in this regard. The purpose is to replicate the transmission of signals through neurons within the same brain region (or cross-regions, boundary conditions abiding) by programming, considering some major simplifications due to the complexity of the real brain. This modelling proposal tackles three fundamental brain properties: metastability, backpropagation and neuroplasticity. For the sake of simplicity, a neuron’s nucleus (soma) will be referred to as a “neuron” from here onward.

In this first introductory section, the scope and objectives of the article are disclosed. In Section 2, the theoretical background and state of the art are presented as the basis of the methodology explained in Section 3. Section 4 showcases varied preliminary results as proof of concept—which will be discussed in Section 5. Lastly, Section 6 contains some broad conclusions drawn out of the article as a whole.

## 2. Modelling Aspects

In this section, some aspects of the model to be introduced below are addressed.

### 2.1. Proposed Neuronal Model and Communitarian Interactions

Neurons are modelled as cells in an agent-based framework. This implies that neurons, as agent-based cells, have communitarian behaviour and interactions and, as biological cells, consume resources and may migrate. Such interactions have an influence over their neighbours—topology, structural and functional connectivity, small-worldness, etc.—measurable through a certain set of parameters associated to each one of them—synaptic weights, activation, thresholds, neighbourhood and sense of information flow. As is typical in agent-based models, neurons behave in an autonomous, unsupervised and proactive manner, as a sort of independent dynamical decision makers [24]. Such models are ubiquitous in fields of science where community interactions are the focus of the study, such as biology or social networks, and well implemented in several commercial software packages [25].

Neurons display connections between them (synapses) and propagate pulses through other neurons. Signal transmission is modelled like the McCulloch–Pitts [26] neuron, but incorporating modifications to include the biological point of view. With this approach, signal processing can be measured and studied incorporating mathematical tools and concepts like convergence or accuracy, but a biological interpretation of the neuron and the incorporation of biological aspects into the full network is also possible.

#### 2.1.1. The McCulloch–Pitts Neuron Model

The McCulloch–Pitts model is the first mathematical model of the signal processing in a biological neuron [26], capable to fire and behave in a similar way to a real neuron. The neuron has connections with a variable number of presynaptic neurons, each one with a different random potential. Once some of the presynaptic neurons have fired, if the average potential received from those neurons is higher than a certain threshold, the neuron will fire as well; otherwise it will stay latent. The main drawback of the McCulloch–Pitts model is the restriction to produce only binary outputs (fire or not-fire). Thus, typical artificial neural networks enhance the capabilities of the McCulloch–Pitts model through the consideration of variable weights and more elaborate firing rules.

In this work, an enhanced McCulloch-Pitts neuron signal processing model is implemented within an agent cell in an agent-based model. This way, neurons process signals while also being capable of performing plastic remodelling, migrating or inhibiting its biological counterparts, dynamically changing the brain processing map by biological interactions, not just by signal weight changes.

#### 2.1.2. Inhibition and Excitation

In the biological model, there are neurons in charge of supervising the community learning. The neurons in charge of the supervised learning of the community can emit inhibitory and excitatory signals, which are propagated through the network via backpropagation. At each iteration of the model, 0.05% of the neurons with a higher signalling threshold for inhibition or excitation are the ones that will change their behaviour in subsequent steps. In the proposed approach, when a neuron enters inhibition, it is modelled by assuming a higher firing threshold, which acts as a switch-off interrupter. The excitation mode is enforced through plastic remodelling and by setting a lower firing threshold.

#### 2.1.3. The Plastic Remodelling Process

In the proposed model, the plastic remodelling process is addressed through changes in the connectivity of a given neuron with its presynaptic neurons: when a neuron receives enough excitatory signalling, the neuron modifies its presynaptic neurons. This is mandated when the neuron in charge of supervising the training process demands to receive a signal earlier than its current latency. Then, the neuron under plastic remodelling looks for new presynaptic neurons between the ones that have fired earlier than its current presynaptic connections. Plastic remodelling is also known as neuroplasticity, which can be structural [27]—rewiring neuronal infrastructure, i.e., “brain’s highways”—and functional, changing the use patterns of said infrastructure, i.e., “neural traffic” [28]. These concepts will be explained in a more detailed manner in Section 2.4.

#### 2.1.4. Migration

In the present model, a third way by which the neurons alter the pulse propagation is migration. Migration is a common phenomenon affecting some types of cells, including neurons and especially—but not exclusively [29]—during brain development [30] and in an abnormal way as a result of illness [29]. Migration follows two main mechanisms: radial, which can be somal translocation—resettlement of the neuron’s nucleus—or glia-guided locomotion—e.g., from the ventricles to the developing cortical area; and tangential—for instance, cortical interneurons migrating from the ventral telencephalon to the cortex [29].

Meanwhile, the migration process—whatever the mechanism—entails two aspects: leading—determining the direction of migration—and trailing—leaving a wake along which new axonal connections are conformed [31]. New neurons may be added to the model, initially placed randomly within the brain and making connections with the surrounding neurons. Future model development will include different cell migration criteria to improve the adaptability of the neurons.

### 2.2. Metastability

Different wave bands in the brain (alpha, beta, mu, etc.) may be positively or negatively correlated among themselves [32], but they are out of phase anyway, and so they have to be processed. Metastability explains how the brain coordinates diverse input signals from multiple receptors in time (neural oscillations, i.e., generated by sensory neurons after receiving stimuli) into a coherent, unison response, locked in frequency (like the movement order sent to a muscle from a motor neuron).

This way, the brain makes sense of all the unsorted, unorganized information it receives to produce meaningful data that will decide a given outcome [33]. These brain waves synchronise, travelling in sequence, and experience nonlinear instabilities [34]. Metastability coordinates the flow of information between brain areas in long spatial and temporal intervals, generating perception, emotion, and ultimately, cognition itself, restarting the latter after sleep [35].

### 2.3. Backpropagation

Neural backpropagation happens from the axon hillock to the dendrites aiming for the dendritic voltage-gated calcium [36] or sodium channels [37] when the soma undergoes depolarization. There is empirical evidence of pyramidal neurons (with separated apical and basal dendrites) performing backpropagation [38] and modelling attempts of this phenomenon are abundant [39,40,41]. Nonetheless, this neurological concept differs greatly from its more widely known mathematical counterpart in Machine Learning, as will be explained in Section 2.5.

### 2.4. Neuroplasticity

Neuroplasticity is the brain’s ability to modify its connections and/or paths so that the information can get across despite severed or malfunctioning connections, avoiding them and finding alternatives. It has been empirically proven that this process takes place in brains of any age, regardless of illness [42]. This process can be structural, by regeneration or collateral sprouting (reactive synaptogenesis, rerouting, retraction [28]); or functional, by task relocation within an already existing neural infrastructure (homologous area adaptation, cross-modal reassignment, map expansion, and compensatory masquerade [27]).

Structural changes like neurogenesis occur almost exclusively in the hippocampus and olfactory bulb [43] and can be enhanced positively (exercise and good environmental conditions), or negatively (stress, injury, disease). Some functional changes such as map expansion are ubiquitous and life-long [27].

Neuroplasticity is ultimately responsible for the adaptation of the brain’s structural and/or functional connections to external-senses- and intrinsic stimuli-learning. This adaptation occurs during growth and after injuries [44], regaining lost functionalities [45], even to astonishing extents in some cases [46]. Diaschisis (or “functional splitting” [47]) is a phenomenon closely related to neuroplasticity, consisting of a sudden function change/inhibition in a brain area caused by disturbance/damage in another distant but structurally connected zone. This happens mainly in severed connections within the Central Nervous System (CNS).

### 2.5. Biological and Artificial Neural Networks

Although artificial neural networks (ANNs) are in fact inspired by the signal processing of their biological counterparts in the brain [26], several authors [48,49,50] have pointed out major differences between them. Thus, the approach presented in this paper is hybrid, introducing Graph Theory and Machine Learning considerations while accounting for real brain phenomena for biomedical accuracy.

In ANNs, the signal propagates forward in the first place. By repeatedly applying the chain rule of derivatives, one can define the rate of change (gradient) of the output prediction $\hat{y}$ or any layer’s immediate outputs $z_{i}$ in relation to a given input $x_{i}$, being $z_{i}=W_{i}x_{i}+b_{i}$. After evaluation of a given loss function $E=f(y,\hat{y})$, backpropagation in ANNs is the process by which signals travel backwards (from outputs towards inputs) in order to correct previous steps and so approach a target output *y*, recomputing the weights $W_{i}$ and biases $b_{i}$ of each neuron layer to “learn” the combination that yields the target outcome. Using Automatic Differentation (AD) [51], that gradient derivation is carried out by most Artificial Intelligence packages such as Pytorch© or Tensorflow©.

A monotonic (ever-growing) activation function is enforced to cast the layer’s output $a_{i}=f\left(z_{i}\right)$ into a [0,1] interval, such as hyperbolic tangent (tanh), rectified linear units (ReLU), exponential (sigmoid) or any of their variants. In the most common ANN architectures, such as MultiLayer Perceptrons (MLP), Convolutional (CNN) or Recurrent Neural Networks (RNN), layers are usually fully connected, meaning all neurons in layer *i* are connected to all their inputs in the previous layer i−1 and all their outputs in the next one, i+1, and the relevance of connections is typically left to the weights, even though in some deep ANN “weak” connections may be eliminated. There are some exceptions like Graph Neural Networks (GNN), in which partially-connected graphs can still have their nodal and edge attributes updated through specific functions instead of directly computing the gradient, a technique known as “Message Passing” [52].

As for the activation functions in biological neurons, membrane activation (responsible for neurons and muscle cells) does have a threshold (an electrical activation potential around −55 mV, which can be graded) but, remarkably, its activation curve is not monotonic, going through separate steps: depolarization ($Na^{+}$ ions enter, potential rising to the maximum, +40 mV), repolarization ($K^{+}$ ions exit, potential decreasing to the minimum), refractory (hyperpolarization) and resting (potential stabilized at −70 mV). Synapses themselves are regulated by very convoluted biochemical (neurotransmitters, proteins) and electrical processes which are usually not considered in a ANN model at all, nor in this proposal.

Such a mathematical reduction must be taken into account to avoid overly simplistic or purely deterministic conclusions or generalizations extracted from the use of ANNs. On top of that, it takes milliseconds for a single synapse to happen [48], a somewhat slow rate if compared to some high-performance multi-layered ANNs after training—which does require longer times, especially in deep networks with convolutions [53].

Conversely, inhibitory and excitatory paths for backpropagation in the real brain are distinct, since neurons produce either inhibitory or excitatory synapses, but not both. Real inhibition/excitation paths are diffuse since biological neurons are not fully connected, let alone layer-structured, and they receive no information other than their preceding neighbours’ outputs [48], which constitutes a direct obstacle to perform backpropagation where all weights are needed, an inconvenience sometimes referred to as the “synaptic assignment problem” [54].

Moreover, neurons in primates tend to activate on the basis of attention mechanisms rather than error backpropagation when reacting to visual stimuli [55]. Some ANN models consider this behaviour [56]. For example, they become active when learning to recognize and classify physical shapes [57].

Synapses between neurons are also subject to plasticity: their intensity and distribution change according to the task [58], electrical stimulation [59], age [60,61] and damage [28,62]. In essence, synapses are also trainable [63]. Indeed, data scientists have suggested more realistic computational approaches accounting for neuroplasticity as a simple given rule [38,49,54,64,65,66,67].

In biological neural networks, Hebb’s learning rule applies: “neurons that fire together, wire together”, implying functional connectivity determines the structural connectivity, so structural plasticity would submit to functional needs. Such a restriction poses a problem for a realistic implementation of ANNs, since neural connections must be strong enough to ease memory recalls but not too strong to create numerical overcharge. Some solutions to this problem imply transient nonlinear analysis [68].

## 3. Methodology

All needed data is produced by self-made code in Python© considering some limitations such as size. Size limitations in the model are needed because the real amount of neurons is estimated to be around 100 billion [69]. The more computing power the modeller has at his/her disposal, the bigger—and more accurate—the model will be. This proposition is in its initial stages, and authors are aware and acknowledge its limitations, but such a limited computational cost can prove an advantage when means are scarce—i.e., emergency situations and/or unavailability of instrumentation.

Moreover, the order of magnitude of total synapses in a young brain is around $10^{15}$, even though they change in number and spatial distribution with age [70,71,72], illness [73,74,75] and lack thereof [76], specific area and chemical procedures. Indeed, mapping the brain is a difficult topic, as previously mentioned. In this work, synapses are considered a quasi-instantaneous, purely electrical process.

In the proposed model, 317,321 synaptic connections and 42 thousand neurons have been used to represent the parts of a logic gate. For comparison, a modern CPU contains roughly 100 million logic gates. The proportion between the neurons for the reproduction of such a logic gate and the ones needed in a future application to reproduce computing capabilities are of the same order.

Out of all neurons used in the model, one thousand are stimulated, and thus in charge of the spike initiation. Another thousand neurons are in charge of supervising the learning of the network. The remaining 40 thousand are intermediate neurons constituting a dynamic network that undergoes structural remodelling. Mathematically, all the neurons are equal, but they have different roles assigned in the network.

A physical domain in $R^{3}$ is created, consisting of a 4×0.5×0.5mm prism. The volume is $1\;{\mathrm{mm}}^{3}$ which reproduces the estimated neuronal density of the brain of 40,000 $\mathrm{neurons}/{\mathrm{mm}}^{3}$ [77]. In two boundaries of the domain (frontal and posterior faces of the prism), $n_{1}$ stimulated and $n_{2}$ supervising neurons are placed. Between these two areas, the number is much higher, $n_{0}=20\times \left(n_{1}+n_{2}\right)$ neurons. The spatial coordinates of these neurons are given randomly within the domain in a sparse pattern. All these neurons represent different global inputs (emitter neurons) and outputs (receptor neurons) and their synapses determine the processing paths between them within a certain region of the brain.

The initial random structural connections are deployed between the neighbouring neurons. Neighbouring connections (neuron spatial map) are defined through Delaunay’s triangulation, generalized to N-dimensions through the Bowyer–Watson’s algorithm [78,79]. This approach is widely used to create unstructured meshes [80], yielding tetrahedra whose vertices are contained within their correspondent circumsphere.

Its homogenizing properties in joint angle and edge (axon) length are useful to accurately represent a network composed of one specific type of neuron, presenting similar though not identical morphology—perhaps belonging to a volumetric sample within the same brain region. This configuration also avoids unforeseen elements in the structure (unwanted neurons or connections between them), since it prevents edge intersection. After this process, the initial structural connectome (the brain’s infrastructure) is established.

Every neuron *i* is given an initial weight $w_{i}\in \left[-1,1\right]$ and an activation threshold $\alpha_{i}$ (initially zero), so that if ${\bar{w}}_{I_{i}}\geq \alpha_{i}$, it will be activated, being $I_{i}$ the set of input neurons of *i* firing at this time step, and ${\bar{w}}_{I_{i}}$ their average value. In a first approach, structural and functional connectivity were considered equivalent, so information would flow always from emitters to receptors.

Nonetheless, neurons are not aligned in the real brain, neither sense-wise (soma to telodendria) nor path-wise (inputs to outputs), so the sense is randomly decided for each of them. The only rule that applies is the non-connectivity of input or output neurons among themselves if they are treated as stimulated and supervising neurons, respectively. Following this reasoning, whichever inputs a given soma (nucleus) receives are considered its dendrites, and so its outputs would correspond to its axon terminals (telodendria), as in multipolar neurons all around the Central Nervous System. Then, edges in this model represent either dendrites or axon terminals, whereas nodes include every other part in the middle (nuclei, axon hillock, axon, myelin sheath, etc.).

Of course, this is not the case in the real brain, where—as explained earlier—neurons are oriented in a given direction and sense: dendrites-soma-axon. The purpose of this randomly assigned sense is to replicate the disorganised orientation of neurons, ignoring—by know—the different biological functions of dendrites and axons as this model focus exclusively on the flow of information throughout neural networks in the brain. Further biochemical implications can—and will—be included in later stages.

To emulate brain metastability, the density distribution of active supervising (or output) neurons must synchronise with a given desired target signal, for instance, an exponential curve centred at a certain propagation step. To train that synchronisation, certain sets (fractions) of input neurons (stimulated, $n_{1}$) are sequentially lit by default during the initial time steps and so the firing of neurons propagates forward, eventually reaching the supervising neurons and activating some of them.

At each step, inhibitory and excitatory signals propagate backwards to neurons with incorrect status, inhibiting the neurons that should have been off but are on and exciting the inactive ones that should have been active. This is the supervised learning mechanism that the $n_{2}$ neurons apply to the neuronal net. After this first training iteration, newly corrected forward propagations are sent to increase accuracy with each step. By the end of a simulation, the more closely the synchronised combined signals approach the target distribution, the more accurate the model will be.

Due to the sense randomness introduced, some signals are effectively lost, as expected, reaching a point where they cannot find a viable path matching the activation conditions and so they disappear. This can indeed be interpreted as a normal consequence of network randomness. Another interpretation is that of signals travelling to other out-of-scope regions of the brain (far-away connectivity). They also account for neuroplasticity, allowing for new paths to be explored in different simulations. To further enhance signal synchronisation, a specific counter is set so that if the hop-distance (number of connections traversed) between two neurons is equal to the difference in time steps, the activation of the precedent one is promoted.

An ad hoc function introduces a percentage of neurons changing status. The purpose is to mimic functional neuroplasticity by searching for the most busy neurons (through which most signal paths go through) and exciting or inhibiting a small set of them accordingly. This excitation/inhibition is modelled by decreasing or increasing their activation potentials and through plastic remodelling.

Such remodelling depends on whether those paths are mostly inhibitory or excitatory, and recreates the variance in neurotransmitter receptors happening in synaptic plasticity [81]. In Graph Theory terms, these neurons (or nodes) are central, since they have the highest degrees (number of neighbours). The fraction of neurons affected by neural plasticity must be limited to <0.05%—provisional percentage due to computing power limitations, to be improved in future versions—of the total number (i.e., 20) at each adaptation step to avoid numerical instabilities, as previously explained.

As for structural neuroplasticity, that is, changing the connectome’s infrastructure by rearranging, severing and/or creating axons; certain damaged neurons over time (due to age, illness, injury or any combination of them) could force reactive synaptogenesis to occur, relocating connections either in the damaged neuron’s neighbourhood (easiest, most straight-forward) or in unexpected far-away places (emulating axon rerouting and/or age-induced density loss, perhaps diaschisis).

One way of achieving that comes in response to backpropagation: negative-weighted neurons could reconnect along inhibitory paths and positive-weighted neurons could do likewise for excitatory ones, relieving or reinforcing them, respectively. That way, each possibly useful pathway is optimized while the rest perish if unneeded. If neural damage does not take place, axon retraction could be put into practice by progressively trimming (a fraction of) the least-used connections, striving for global connectome efficiency—a process commonly known as “pruning” in neuroscience [82].

Also, neuronal migration is implemented in the model. In this case, new neurons are randomly emerging in the net and connecting with the transition ones. This appearance is also limited to 0.05% of the total number of neurons at each adaptation step—again, to avoid numerical overcharge, but this fraction can be increased if more powerful computers were to be used for more ambitious (realistic) simulations.

For instance, should the modeller want to replicate GABAergic migration in early development, this would affect around 20% of neurons travelling from to ventral telencephalon to the cortex, inhibitory and excitatory in similar proportions [83]. This becomes unbearable with limited computing power. This humble 0.05% percentage could be interpreted as a specific functionally-driven migration rather than a full-scale one—as in developmental brain growth.

The signal initiates with a stimulation of $n_{1}$ neurons in different steps. At the beginning of the calculation, 25% of those neurons fire, in the next propagation step an additional 50% fires, and so does the remaining 25% in the last step. This is, of course, an example of metastability at work—reorganizing a spiking cascade into a coordinated output signal.

**Figure 1:**
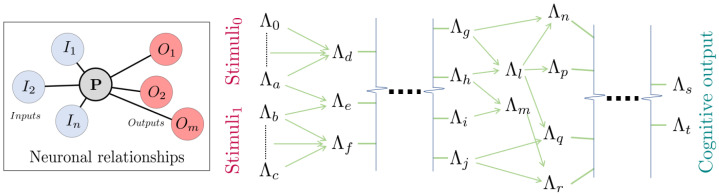
Relationship between inputs and outputs in a modelled neuron (**left**). And concatenation of Λ blocks of neurons to produce complex cognitive outputs (**right**). An example of Λ block including Boolean logic gates (see Figure 2) is used in Figure 3 to modulate the signal.

This way, when the last fraction of $n_{1}$ neurons fires, the signal originated with the first stimulated ones will be 2 propagation steps ahead. Then, the signal is calculated through all the propagation steps from the firing of the $n_{1}$ neurons to the firing of the $n_{2}$ ones. The network is self-remodelled, and a new cycle starts with a new propagation in an iterative process. A workflow visualization can be found in Figure 1.

### 3.1. Creation of Logic Gates with Neurons: Modification of the McCulloch–Pitts Boolean Model

**Figure 2:**
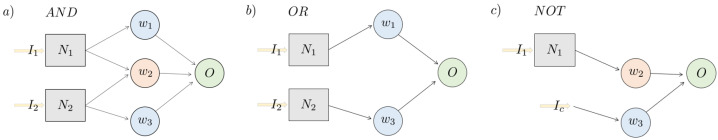
Diagrams of Boolean logic gates for asynchronous stimuli using the proposed methodology: AND (**a**), OR (**b**) and NOT (**c**).

The model shown in the previous section is showcased in the squares of Figure 2, as $N_{1}$ and $N_{2}$. Their role is that, given two stimuli $I_{1}$ and $I_{2}$, not necessarily coordinated, those will be synchronised through the networks $N_{1}$ and $N_{2}$ as validated before. This section is intended to study Boolean configurations in which this model can reproduce logic gates from asynchronous stimuli. The Boolean analogy is fire (true) and not fire (false). Figure 2 shows three schematics for AND, OR and NOT gates.

Those gates, along with their elements, are explained below.

#### 3.1.1. AND Gate

In this case, $w_{1}>0$, $w_{3}=w_{1}$, $w_{2}<-w_{1}$, and the α of the neuron *O* is 0. If $I_{1}$ or $I_{2}$ are stimulated alone, the average $\bar{w}$ is lower than the threshold α, so that only if both fire at the same time, $\bar{w}>\alpha$ holds true. Of course, any other combination of values for those variables can achieve the same objective as long as those proportions are fulfilled.

The Boolean output (*O*) can be defined as $O=\;I_{1}\;AND\;I_{2}$, if there is a stimulation in $I_{1}$ and $I_{2}$ the output in *O* is to fire, otherwise *O* would not fire.

#### 3.1.2. OR Gate

For this gate, any stimulus $I_{1}$ or $I_{2}$ will make *O* fire, since the inhibitory intermediate neuron with $w_{2}$ is removed. The Boolean output (*O*) can be defined as $O=\;I_{1}\;or\;I_{2}$ in this case.

#### 3.1.3. NOT Gate

This logic gate is intended to switch between the state of $I_{1}$ and *O*. Here, only one input is required, but a continuous fire $I_{c}$ is needed to ensure that, when $I_{1}$ is not stimulated, *O* is. For this reason, a constant firing signal is needed. when $I_{1}$ is stimulated, the inhibitory neuron $w_{2}$ is activated and so *O* does not fire, since now $\bar{w}<\alpha$. The Boolean output can be defined as $O=\;NOT\;I_{1}$.

## 4. Results

This section shows some results of the implemented model. Regarding computational times, in a serial code in Python, run on a single core, a simulation took 43 s. For every adaptation step of the neuronal network, 24 iterations were needed in total to adapt the network in the two cases. As mentioned before, the adaptation process is two-fold: logical and biophysical. The logical side consists of inhibition and excitation that result in plastic remodelling. The biophysical part involves migration of new neurons to the area. The results show the network’s capability to adapt the delay of a signal when subjected to change, which is essential for the logic gate models proposed.

### Decrease and Increase in Pulse Latency

**Figure 3:**
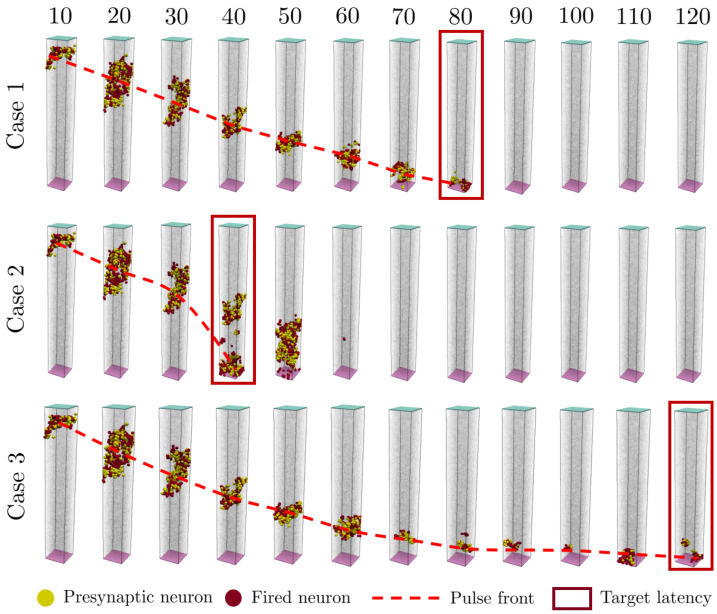
Signal progression in the three cases studied: reference (Case 1), reduced delay (Case 2) and increased delay (Case 3). Note that the reference case has an almost constant propagation rate, whereas the other two cases modify the signal propagation rates.

In this case, with the model described in the methodology, the delay of a pulse is increased and decreased from the reference latency of the network. The reference latency is 80 propagation steps (Case 1 of Figure 3, and the target is to reduce it to 40 (Case 2) and to increase it to 120 (Case 3). Considering that the length of the prism studied is 4 mm, and that it has a reference latency of 80 propagation steps, the signal advances as an average 0.05 mm for each propagation step. The speed of the signal in the brain can range from 0.5 to 100 m/s, which implies that considering, for example, 10 m/s as the reference velocity, each propagation step will be equal to 5 μs.

In Figure 3, the top square shown in every lapse (coloured in light blue) represents neurons excited with the predefined input signal. The bottom square (coloured in light purple) is where the supervising neurons are. So the signal goes from the top to the bottom, and once the supervising neurons get excited, they send backpropagation signals according to their target signal delay.

Figure 3 shows the firing steps of the adapted networks. Case 1 shows the firing steps of the reference network, the one generated randomly as explained in the methodology: signal arrival takes place at 80 propagation steps. This case 1 is also the starting point for the remodelling of the network to achieve the states of Case 2 and Case 3. Case 2 is the result after remodelling to reduce the latency of the network, e.g., signal arrival takes place at 40 propagation steps.

Also in Figure 3, the red dashed line shows that the front progress velocity is not constant: at the end of Case 2 it is accelerated, whereas Case 3 ends with a reduced speed. In Case 1, this propagation velocity is roughly constant as a consequence of the equally random distribution of the neurons and their synapses.

In Case 2 of Figure 3, at propagation step 50 it can be seen that the signal front goes backwards and intercepts other signal fronts in the neuronal network. Those are spurious signals that are naturally diminished in the model before reaching the output.

**Figure 4:**
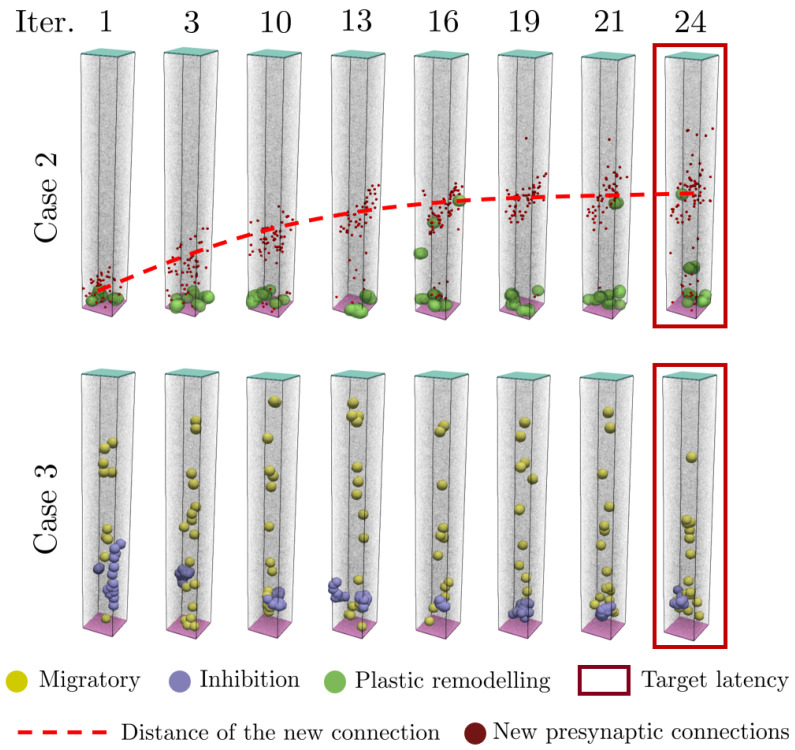
Remodelling strategies adopted by the neurons for Cases 2 and 3 during progressive iterations to achieve the target latency.

The results shown in Figure 3 have been obtained after the remodelling process. Figure 4 shows the changes involved in the inhibition and excitation needed to change the delay of the signal for Cases 2 and 3. In Case 2, it is observed that changes are mainly due to plastic remodelling (variations in synaptic connections, new presynaptic connections), and with every iteration the new and the average distance of the connected neurons increase. After a given number of iterations, the neural structure converges. In Case 3, synaptic plastic remodelling does not seem to be sufficient or adequate, so neurons enter in inhibitory or migratory status to achieve a delay in the requested latency of the signal mandated by supervisory neurons.

In the Case 2 of Figure 4, plastic remodelling of the neurons allows the signal to go faster through the network. As mentioned in the methodology, such remodelling is carried out by changing the synapses. The red dashed line shows that, as the plastic remodelling progresses, the synaptic connections are wired at a higher distance.

In Case 3 of Figure 4, the signal arrival takes place at 120 propagation steps, showing the remodelling carried out by the network to increase the latency of the system. Here, some neurons are inhibited to extend the path of the signal and other neurons migrate and connect with the network to prevent signal blackout and ensure its continuity.

**Figure 5:**
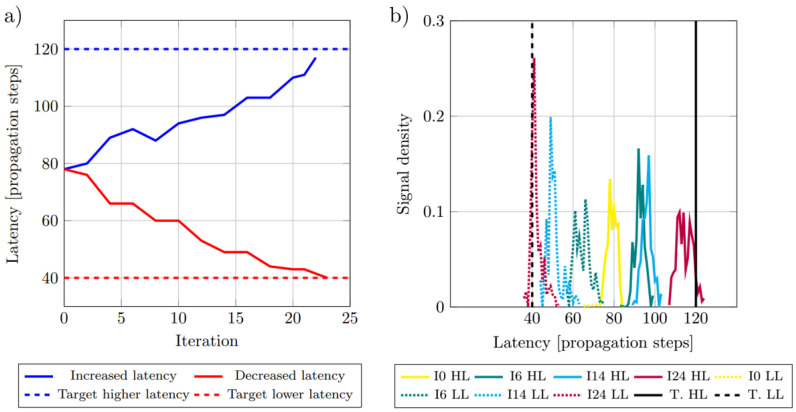
Evolution of the output signal after remodelling iterations. (**a**): Evolution of the latency of the signal towards the target with iterations. Faster convergence to the decreased latency can be observed, due to neural migration phenomena. (**b**): Evolution of the received signal density with propagation steps, during the iterations. H (continuous) and L (dashed) stand for higher and lower latency, respectively, T for target and I for iteration. Note how the signal generally tends to showcase higher amplitudes (spikes) for lower latencies, due to the high number (and thus, density) of neurons (migrating or not) involved in shortening the path.

In quantitative terms, Figure 5a shows the evolution of the signal’s delay for different remodelling iterations until they reach the target. Figure 5b displays the output signal at different remodelling iterations for both the increased and decreased delays.

This proves that with two of those prisms in parallel, different uncoordinated stimuli originated in different parts can result in a coordinated pair of signals—i.e., brain metastability—capable to perform logic calculations. Further explanations are offered in the next section.

## 5. Discussion

The results reflect a simplified yet sufficiently realistic representation of the brain’s information flow considering the low computational cost: it imitates metastability by coordinating one or multiple diverse and out-of-phase input signals into a single output target signal, while enforcing biologically plausible backpropagation with separate inhibitory and excitatory paths—further enhanced by the introduced neuroplasticity functions. It must be mentioned that this model reaches the target by organically adjusting functional and structural paths, without any explicit optimization parameters nor loss functions.

Once again, authors would like to acknowledge its limitations regarding biological and chemical nuances not captured by this preliminary approach, which, of course, have a tangible influence in the brain’s function, including information flow, around which this whole article revolves. This is but a first attempt in replicating complex brain phenomena—metastability, neural backpropagation, neuroplasticity and neuronal migration—from a mathematical perspective with some simple biological considerations. The intention is that this model becomes a cornerstone for more complex and complete versions including, among others, biological and physiological damage, neurochemistry, multi-scale and nonlinear considerations.

In spite of these satisfactory results, the authors are aware of this model’s weaknesses, especially regarding the step around which the target signal is centered. Focusing solely on information flow through the network, some limitations become apparent, namely the shortest/longest path problem.

On the one hand, moving the target “backwards” in time (fewer propagation steps) is equivalent to organically shortening paths to arrive to the same output. Computationally speaking, for a negatively-weighted, undirected partially connected graph like this; such a problem can be solved by algorithms such as Bellman–Ford’s [84], Johnson’s [85] or Floyd–Warshall’s ([86], worst-case complexity $O(V^{3})$, being $V=n_{0}+n_{1}+n_{2}$ the total number of connected vertices). Obviously, it is unlikely that this network heuristically solves such a problem in a limited amount of steps, although it can be helped through neural migration to a certain extent.

On the other hand, should the target move “forward” in time (more steps), the model would have to artificially extend paths to synchronise the signals, searching for the longest path, which constitutes a NP-hard problem—escalating to NP-complete if a certain length is sought. Some solutions exist for directed, acyclic graphs [87,88] (even with perturbations—adding and eliminating edges—[89]), but checking whether a Delaunay-generated graph contains cycles is a NP-complete problem by itself [90,91].

As a clarifying note, NP-hard problems are those that have a binary answer (yes/no) and whose individual cases—but not general solutions, which may well not exist—can be checked within a polynomial—meaning finite—time. NP-complete is a subset of NP-hard problems concerning the “translation” of one NP-hard problem into another one in polynomial time—which is still more difficult to prove. Understandably, the emergence of such problems within a model poses severe practical issues, mainly related to reasonable computing times.

Migration mitigates these issues to some degree by relocating neurons—albeit a tiny fraction of them (0.05%) to keep computational costs relatively low—throughout the network and thus organically shortening some paths and enlarging others. To further address this issue, some global (clustering, small-world coefficients) and node-dependent (vulnerability, shortest path) graph parameters could be introduced and leveraged to choose the most convenient path—short or long, depending on the target signal’s position in time.

Such indicators would deliver useful information on the topological characteristics of the connectome, so that certain clustered areas—known as “hubs” [92]—could be avoided (shorter paths) or crossed (longer paths), whereas vulnerability-related indicators could provide very valuable information on which alternative paths to follow when certain connections (structural or functional) are damaged or even severed—neuroplasticity in practice. They have already been studied on a brain regional level [93,94,95,96], but not on a neuron level like suggested here.

A graph-based approach entails two self-evident ramifications for brain networks: the multi-scale approach—in both time [97] and space [98] and the involvement of Graph Neural Networks [99,100,101,102,103] or similarly-flavoured neural network techniques [104,105]—notwithstanding the aforementioned caveats.

Another option would be to directly introduce rules to shorten or enlarge neural pathways if the target gets closer or further, respectively, although biological evidence for such a behaviour remains elusive. Introducing time constraints, such as refractory periods [64] replicating membrane functioning could help achieve this goal, since the present approach does not considerchronological time but rather propagation steps (quasi-static). In this scenario, neuron length does not quite play a practical role, so the only meaningful way to shorten (or stretch) neural paths would be to artificially skip (or add) synapses along the way.

The next significant step to be taken is the development of a bio-mechanical model of the brain which reflects the interaction between information transmission and external loads or accelerations (leading to Traumatic Brain Injury), loss or damage of axons (neurodegenerative diseases) or even areas with distinct properties (tumour growth, brain stiffening caused by Alzheimer’s).

There is a plethora of bibliography proposing bio-mechanical models [106,107,108,109,110,111,112,113,114,115], but the correlation between physical damage and information flow leaves room for research. Also, the model itself could be enriched by including and emphasizing the role of other parts of the neuron (like the myelin sheath) or another components of the CNS, such as glial cells [116].

Bearing all these suggestions in mind, further developments of this code are being studied, aiming at a better-performing model with bio-mechanical and chemical implications.

## 6. Conclusions

In this work, some aspects of the modelling of brain plasticity have been addressed. Remodelling the brain implies both logical (presynaptic connections and signal processing strengths) and biophysical considerations (cell migration, community behaviour, inhibition and excitation, among others). The relevance of these features has been put to the test regarding brain plasticity, in particular remodelling to reach target signal latencies.

The results are promising since they illustrate how computationally affordable simulations can somewhat convey complex brain phenomena—albeit with simplifications—and motivate further research to better understand changes in brain processing, like those related to ageing, illness and/or injuries—soon to be explored in future updates of this work.

The present model is also capable of reproducing the Boolean logic behaviour of neuronal communities. This is achieved through the biological rules for interaction, considering neurons as cells able to perform synaptic connections in order to transfer information through signalling. This capability of the model is validated by the two given examples where the latency of a signal through a neuronal community is delayed or increased.

From the presented results, we can infer that, despite the almost unfathomable complexity of the human brain, some of its functions—namely, migration, neuroplasticity, backpropagation and metastability—can be replicated in a qualitative manner, albeit rudimentary. Quantitative verification, for the time being, remains difficult due to the scarcity of such experimental tests, although agent-based modelling of functional brain connectivity appears to be a hot research topic [117,118], even in combination with Machine Learning techniques like in our model [119].

The high ratio between the flexibility and fidelity of our model and the computational cost undertaken to obtain them is relevant to one of computational neuroscience’s biggest issues, besides the already mentioned reproducibility problem. Computationally affordable simulations like ours are vital for the spread of medical monitoring of the brain to every corner of the world, especially in low-income countries where cheap alternatives to costly and unavailable equipment are most needed.

New upcoming developments of this model will strive for a better, more precise portrayal of the brain in multiple scales, both in time and space, and incorporating damage so it can be used by physicians for preventive diagnosis, follow-up and treatment of brain illnesses and injuries on an individualized basis—ideally yielding a so-called “Digital Twin” of each patient’s brain.

## Data Availability

Dataset available on request from the authors.
